# Caring for older adults in the Emergency Department: a Delphi-based national consensus by the Academy of Emergency Medicine and Care and the Italian Society of Gerontology and Geriatrics

**DOI:** 10.1007/s41999-026-01462-6

**Published:** 2026-05-03

**Authors:** Maria Cristina Ferrara, Eleonora Cucini, Martina Marelli, Antonella Zambon, Andrea Ungar, Lorenzo Ghiadoni, Antonio Cherubini, Michele Cosimo Santoro, Alessandra Marengoni, Ciro Paolillo, Ivo Casagranda, Giuseppe Bellelli, Expert Panel, Ernesto Contro, Paola Vignola, Mara Rosada, Fabio Malalan, Gabriele Farina, Fabrizio Giostra, Francesco Franceschi, Simone Magazzini, Eleonora Arboscello, Filippo Numeroso, Paolo Pinnaparpaglia, Vito Cianci, Paolo Groff, Diego Paternosto, Fulvio Morello, Laura Orlandini, Mario Bo, Stefano Volpato, Antonella Mandas, Chiara Mussi, Francesco Landi, Giovambattista Desideri, Enrico Benvenuti, Marcello Maggio, Andrea Corsonello, Angela Sciacqua, Anna Casanova, Agostino Virdis

**Affiliations:** 1 School of Medicine and Surgery, University of Milano-Bicocca, Piazza Dell’Ateneo Nuovo, 1 - 20126, Milan, Italy; 2 Centro Studi Dipartimentale sulla Medicina della Complessità e Cure Palliative Virgilio Floriani, University of Milano-Bicocca, Monza, Italy; 3 Department of Statistics and Quantitative Methods, University of Milano-Bicocca, Milan, Italy; 4 Biostatistics Unit, IRCCS Istituto Auxologico Italiano, Milan, Italy; 5 Geriatric and Cardio-Geriatric Medicine, Department of Clinical and Experimental Medicine, University of Florence and Careggi Hospital, Florence, Italy; 6 Department of Clinical and Experimental Medicine, University of Pisa, Pisa, Italy; 7 Department of Clinical and Molecular Sciences, Università Politecnica delle Marche, Ancona, Italy; 8 Geriatria, Accettazione geriatrica e Centro di Ricerca per l’invecchiamento, IRCCS INRCA, Ancona, Italy; 9 Emergency Department, Foundation Polyclinic University A. Gemelli, Rome, Italy; 10 Department of Clinical and Experimental Science, University of Brescia, Brescia, Italy; 11 Aging Research Center, Department of NVS, Karolinska Institutet, Stockholm, Sweden; 12 Emergency Department, Ospedale Civile Maggiore AOUI Verona, Verona, Italy; 13 Academy of Emergency Medicine and Care (AcEMC), Pavia, Italy; 14 Acute Geriatric Unit, Fondazione IRCCS San Gerardo dei Tintori, Monza, Italy

**Keywords:** Emergency Department, Older adults, Observation Unit, Delphi consensus

## Abstract

**Purpose:**

The number of older adults attending Emergency Departments (EDs) is increasing rapidly in Italy. This trend poses substantial challenges to services that are not structured to address multidimensional needs. Despite the availability of international guidance, implementation of geriatric emergency care models remains inconsistent in Italy. This study aimed to establish a national, inter-specialty expert consensus on priorities for improving geriatric emergency care.

**Methods:**

A two-round Delphi process was conducted involving 32 experts (16 geriatricians and 16 emergency physicians) from two Italian Academic Societies: the Academy of Emergency Medicine and Care, and the Italian Society of Gerontology and Geriatrics. Participants rated 50 items, grouped into 10 statements covering triage, infrastructure, organization, clinical priorities, and care models, using a 5-point Likert scale. Consensus was defined as ≥ 80% agreement or disagreement for each item.

**Results:**

Consensus was achieved for 37 of 50 items (74%). Panelists agreed on the need for early identification of frailty and delirium, environmental adaptations within EDs, and multidisciplinary management in ED Observation Units. Disagreement was observed regarding triage models for older adults, the mode of geriatrician involvement in the ED (on-site vs on-call), and staff perceptions on the appropriateness of ED use by older adults.

**Conclusion:**

This national consensus defines priorities for geriatric emergency care in Italy, while highlighting inter-specialty areas of disagreement. The findings provide a basis for strengthening clinicians’ ability to address complex needs and informing the development of geriatric-oriented ED pathways and policies.

**Graphical abstract:**

**Supplementary Information:**

The online version contains supplementary material available at https://doi.org/10.1007/s41999-026-01462-6.

## Key summary points

**Aim:**

To establish a national expert consensus on priorities and strategies to enhance geriatric emergency care in Italy.

**Findings:**

Consensus was reached on 37 of 50 items (74%), with key agreement on clinical, organizational and environmental adaptations. Divergence persisted concerning triage models, the mode of geriatrician involvement in the ED (on-site vs on-call), and staff perceptions on the appropriateness of ED use by older adults.

**Message:**

This inter-specialty consensus defines priorities for geriatric emergency care in Italy, offering a nationally grounded framework to guide the development of geriatric-oriented ED pathways and policies.

**Supplementary Information:**

The online version contains supplementary material available at https://doi.org/10.1007/s41999-026-01462-6.

## Introduction

The rising number of older adults attending Emergency Departments (EDs) poses substantial challenges to services that are often not structured to address multidimensional needs [1–3].

In Italy, annual ED admissions exceed 20 million. Over the last decade, the proportion of patients aged ≥ 80 years in EDs has risen from 23 to 27%, with visit rates increasing from 335.6 per 1000 inhabitants in the general population to over 650 per 1,000 among individuals aged 90–94 [4, 5]. Moreover, patients aged ≥ 75 accounted for nearly 40% of high-priority cases, underscoring greater clinical urgency and complexity [5–7]. This pattern aligns with international trends, confirming the ED’s expanding role as a critical access point for older adults with unmet needs [8–10].

Managing these patients requires timely, comprehensive assessment to guide appropriate interventions [11, 12]. However, traditional ED models often struggle to provide this approach due to scarce resources, time constraints and limited geriatric knowledge [7, 13]. Clinicians are frequently untrained in recognizing frailty, delirium or other atypical presentations, and standardized protocols for geriatric syndromes remain scarce or inconsistently applied [13–16].

Internationally, structured geriatric emergency initiatives have emerged, and collaborative models that integrate geriatric expertise into the ED have been associated with improved diagnostic accuracy, continuity of care, and patient safety [11, 12, 17–20]. In 2022, the European Task Force on Geriatric Emergency Medicine developed recommendations for the appropriate management of older adults in the ED, encompassing systematic frailty assessment, delirium prevention and management, interdisciplinary care approaches, and age-friendly environmental adaptations [15]. However, Italy was not represented in the European Task Force, and the group’s operational posters are currently unavailable in Italian.

Consequently, routine ED care in Italy remains largely non–geriatric-oriented, with limited use of standardized screening tools for frailty and cognitive vulnerabilities, insufficient integration between emergency physicians and geriatricians, and marked regional heterogeneity. Although sporadic local experiences have been reported—such as the geriatric ED model in Ancona [21], the Frailty Network at Policlinico Gemelli in Rome [22], and integrated pathways like GIROT in Florence [23]—these remain isolated initiatives, often driven by local leadership rather than supported by a national policy framework.

This systemic implementation gap has not been mapped at a national level, but it likely reflects broader structural determinants (historical separation between emergency medicine and geriatrics, persistent ED understaffing, rigid funding models, regional resource disparities) and the lack of national interdisciplinary guidance. This contrasts with recent Italian health policy reforms promoting hospital–community integration for the management of older adults (DM 77/2022 [24]), and underscores the need for scalable models of acute geriatric care.

Within this context, the Academy of Emergency Medicine and Care (AcEMC) and the Italian Society of Gerontology and Geriatrics (SIGG) jointly launched a national Delphi process to define shared priorities for improving geriatric emergency care in Italy. The initiative was designed to identify both areas of agreement and disagreement between geriatricians and emergency physicians in Italy, as a preliminary step toward context-specific implementation of the internationally recommended models.

## Methods

The Delphi consensus approach is a structured, iterative method designed to converge expert opinion through multiple questionnaire rounds, particularly valuable when empirical evidence is limited or expert judgment is essential [25]. Key features include anonymity, controlled feedback and statistical response aggregation, enabling participants to reconsider their positions based on group insights and fostering consensus development [25].

A Steering Committee composed of four senior members from AcEMC and four from SIGG defined ten thematic domains of geriatric emergency care (statements) and drafted a structured 50-item questionnaire. The Delphi expert panel included 32 clinicians (16 geriatricians and 16 emergency physicians). Experts were purposively recruited according to predefined criteria: (i) ≥ 10 years of clinical experience in acute geriatric care and/or EDs; and (ii) a recognized professional profile, as evidenced by formal university-level academic roles, leadership positions in relevant clinical services, and/or peer-reviewed publications in geriatric emergency care. To ensure national representativeness, participants were selected from hospitals throughout Italy hosting both Acute Geriatric Units and Level I–II EDs (see Supplementary File 1—Online Resource S1 for panel details).

An independent methodological expert (AZ) supervised the process and performed the data analysis.

The questionnaire included ten core statements, each articulated into five items (see Supplementary File 1—Online Resource S2), addressing the following areas:

1. Triage procedures
2. ED infrastructure
3. Organizational models
4. Early recognition of clinical priorities
5. Preventive measures to minimize adverse outcomes
6. Strategies to reduce ED length of stay
7. Profiles of patients indicating difficult management
8. Systematic identification of vulnerable older adults
9. Staff perceptions on ED use by older adults
10. Organization of Emergency Department Observation Unit (EDOU)

Item responses were rated on a 5-point Likert scale (1 = strongly disagree, 2 = disagree, 3 = neither disagree nor agree, 4 = agree, 5 = strongly agree). Consensus for each item was defined as ≥ 80% of respondents selecting agreement (scores 4–5) or disagreement (scores 1–2), consistent with Delphi standards [25].

Two Delphi rounds were conducted between July 2024 and January 2025, with full participation.

Anonymity was maintained throughout the Delphi process using a web-based questionnaire platform that concealed participants’ identities. After Round 1, panelists received standardized aggregate feedback for each item, including response distribution and the percent agreement/disagreement, with a clear indication of whether the pre-specified 80% consensus threshold had been reached. Items not achieving consensus were carried forward unchanged to Round 2 and accompanied by brief Steering Committee clarifications to support consistent interpretation (Online Resource S2).

At the end of Round 2, consensus was achieved for the majority of items, with overall agreement exceeding the 70% threshold commonly accepted in Delphi studies [25].

## Results

**Fig. 1:**
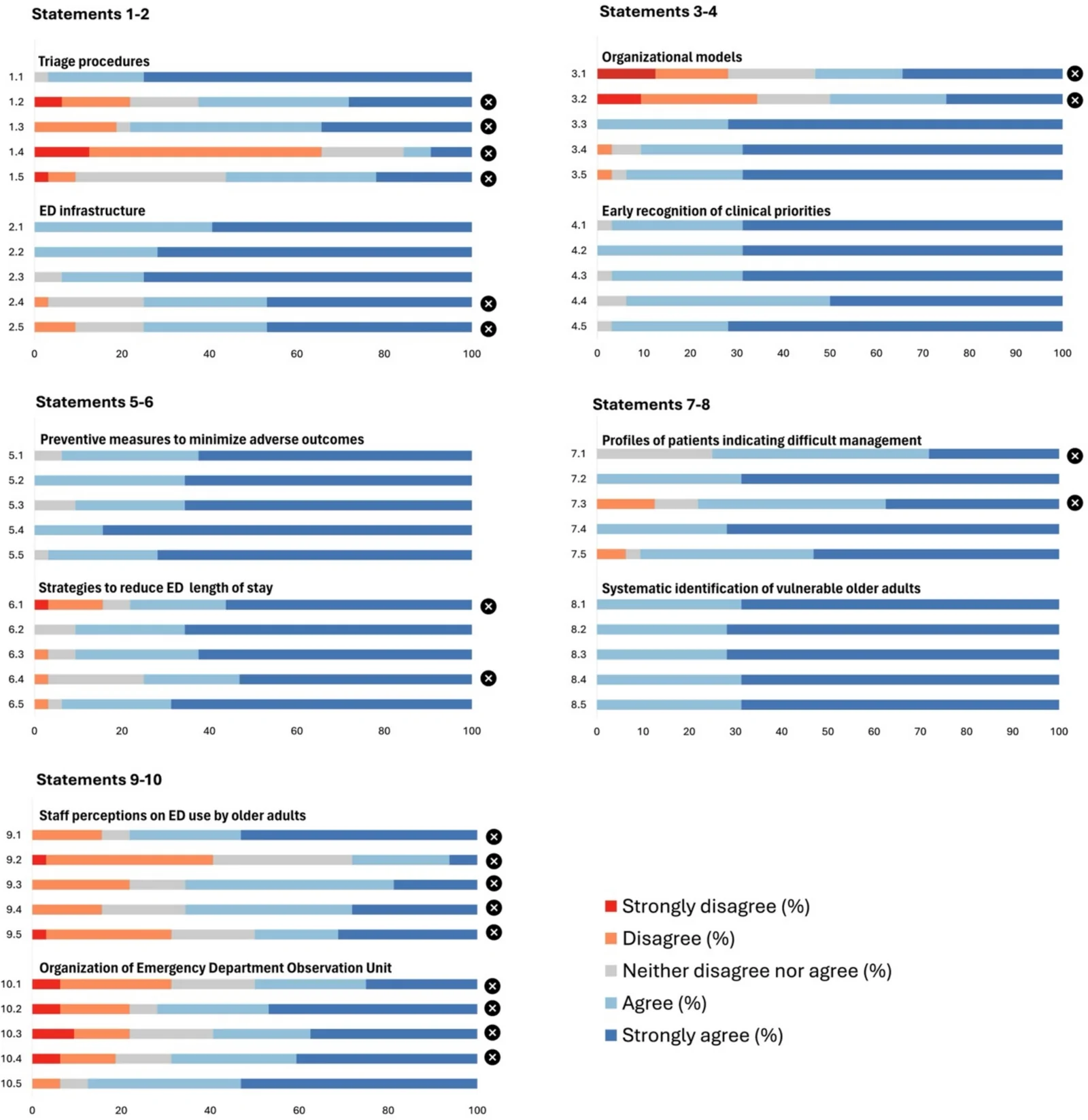
Likert scale responses by item (Round 1). The black cross symbol denotes the absence of consensus among the expert panel for the respective item

Table 1 details consensus status for each of the 50 items across both rounds, including items with persistent disagreement. In Round 1, consensus was reached on 29 items (58%), with unanimous agreement on Statements 4, 5, and 8 (Fig. 1). Key agreements focused on: (i) early identification of at-risk patients, particularly those with atypical presentations of acute illnesses, through systematic assessment of delirium and adverse drug reactions recognition; (ii) targeted interventions to mitigate adverse outcomes during ED stay, through hydration and nutrition protocols, fall prevention, mobility support, dementia-specific pain assessment; (iii) routine identification of patients with frailty, cognitive impairment, social issues, or frequent ED attendance, using specific tools.

**Table 1:** Consensus levels for Delphi statements and items (Rounds 1 and 2)

Statements and items	Consensus levels (%)	
	Round 1	Round 2
**(1) Triage should be structured to consider the characteristics of older patients**		
1.1 Provide specific training on older adults for nurses trained to perform triage	**96.9% (agreement)**	–
1.2 Assign all older adults a Silver Code in addition to the traditional triage code	62.2% (disagreement)	**81.3% (agreement)**
1.3 Direct most patients to a fast-track geriatric pathway	78.1% (disagreement)	65.6% (disagreement)
1.4 Use the “color code” as the sole criterion to determine priority of access	65.6% (disagreement)	68.8% (disagreement)
1.5 Use a triage protocol such as Emergency Severity Index (ESI)	56.3% (disagreement)	50% (disagreement)
**(2) ED architecture should be suitable for the needs of older patients**		
2.1 Provide a waiting/stay area properly equipped for the frailest patients	**100% (agreement)**	–
2.2 Provide space for caregiver presence	**100% (agreement)**	–
2.3 Provide at least movable partitions	**93.8% (agreement)**	–
2.4 Provide an area near the general ED	75% (disagreement)	**81.3% (agreement)**
2.5 Ensure the presence of a geriatric admission room	75% (disagreement)	**81.3% (agreement)**
**(3) The ED should be organized to provide specific management for older patients**		
3.1 Ensure a dedicated presence of a geriatrician in the ED	53.1% (disagreement)	53.2% (disagreement)
3.2 Ensure geriatrician presence only “on-call” for the emergency physician	50% (disagreement)	43.8% (disagreement)
3.3 Train emergency physicians and nurses in managing older patients	**100% (agreement)**	–
3.4 Establish a “frailty unit” with a multidisciplinary approach (emergency physician and nurse, geriatrician, social worker) operating permanently in the ED	**90.6% (agreement)**	–
3.5 Promote constant caregiver presence in the ED	**93.8% (agreement)**	–
**(4) In the general clinical management of older adults in the ED, some priorities should be considered**		
4.1 Consider situations that may indicate a risk of deterioration	**96.9% (agreement)**	–
4.2 Highlight clinical conditions at risk of deterioration with atypical presentation symptoms	**100% (agreement)**	–
4.3 Early identification of patients with delirium using dedicated screening tests	**96.9% (agreement)**	–
4.4 Systematically assess possible adverse drug reactions	**93.8% (agreement)**	–
4.5 Systematically assess the presence of disability and frailty	**96.9% (agreement)**	–
**(5) Interventions should be implemented to reduce the risk of a negative outcome**		
5.1 Provide specific protocols for hydration and nutrition of older adults during their stay in the ED	**93.7% (agreement)**	–
5.2 Systematically identify patients with delirium using specific screening tools	**100% (agreement)**	–
5.3 Implement measures to counteract immobilization	**90.6% (agreement)**	–
5.4 Implement protocols for fall prevention in at-risk patients	**100% (agreement)**	–
5.5 Use pain assessment tools specific for patients with dementia	**96.9% (agreement)**	–
**(6) Interventions should be implemented to reduce the length of stay of older adults in the ED**		
6.1 Increase hospital beds for frail older adults affected by acute conditions	78.1% (disagreement)	**87.5% (agreement)**
6.2 Develop specific clinical pathways for older adults	**90.6% (agreement)**	–
6.3 Ensure the presence of a geriatrician in the ED or on-call	**90.6% (agreement)**	–
6.4 Use the geriatric admission room	75% (disagreement)	**84.4% (agreement)**
6.5 Activate care pathways already agreed with the TOC from the ED	**93.8% (agreement)**	–
**(7) Conditions that suggest overall challenging management in the ED**		
7.1 Older adults > 85 years	75% (disagreement)	**93.8% (agreement)**
7.2 Patients with socio-assistance issues	**100% (agreement)**	–
7.3 Patients coming from nursing homes / protected residences	78.1% (disagreement)	**90.6% (agreement)**
7.4 Patients with significant cognitive deficits and/or behavioral disorders	**100% (agreement)**	–
7.5 Patients with severe motor impairment	**90.6% (agreement)**	–
**(8) Systematic actions should be implemented for proper management of older adults in the ED**		
8.1 Identify frail and/or disabled patients using specific tools	**100% (agreement)**	–
8.2 Identify patients with delirium using specific tools	**100% (agreement)**	–
8.3 Identify patients with pre-existing cognitive deficits	**100% (agreement)**	–
8.4 Identify patients with socio-assistance issues	**100% (agreement)**	–
8.5 Identify patients with repeated ED visits	**100% (agreement)**	–
**(9) Staff perceptions on older adults’ use of the ED can be represented by the following statements**		
9.1 Older patients visit the ED more frequently than younger patients	78.1% (disagreement)	**84.4% (agreement)**
9.2 They are treated with the same intensity of care as younger patients	40.6% (disagreement)	50.2% (disagreement)
9.3 ED use is often inappropriate	65.6% (disagreement)	71.9% (agreement)
9.4 They contribute to overcrowding	65.6% (disagreement)	65.7% (disagreement)
9.5 ED staff should focus on managing critically ill patients, not older adults with chronic conditions	50% (disagreement)	59.4% (disagreement)
**(10) The benefits of an Emergency Department Observation Unit (EDOU) include reduced ED length of stay for older adults, improved ED efficiency, and better clinical and organizational outcomes**		
10.1 Older adults should be admitted exclusively to a dedicated EDOU	50% (disagreement)	53.1% (disagreement)
10.2 The EDOU should include a section reserved for older adults	71.9% (disagreement)	78.1% (disagreement)
10.3 The EDOU should also be managed by geriatricians	59.4% (disagreement)	62.6% (disagreement)
10.4 The EDOU should be managed by emergency physicians	68.8% (disagreement)	53.1% (disagreement)
10.5 The EDOU should be managed with a multidisciplinary approach (emergency physician + geriatrician)	**87.5% (agreement)**	–

The table reports the percentages of consensus achieved for each item at the conclusion of each round. Cells highlighted in orange with a black cross indicate a lack of consensus for the global statement (i.e., across all items). Cells highlighted in green with a checkmark indicate that consensus was achieved for all items within the statement at either the first or second round

**Fig. 2:**
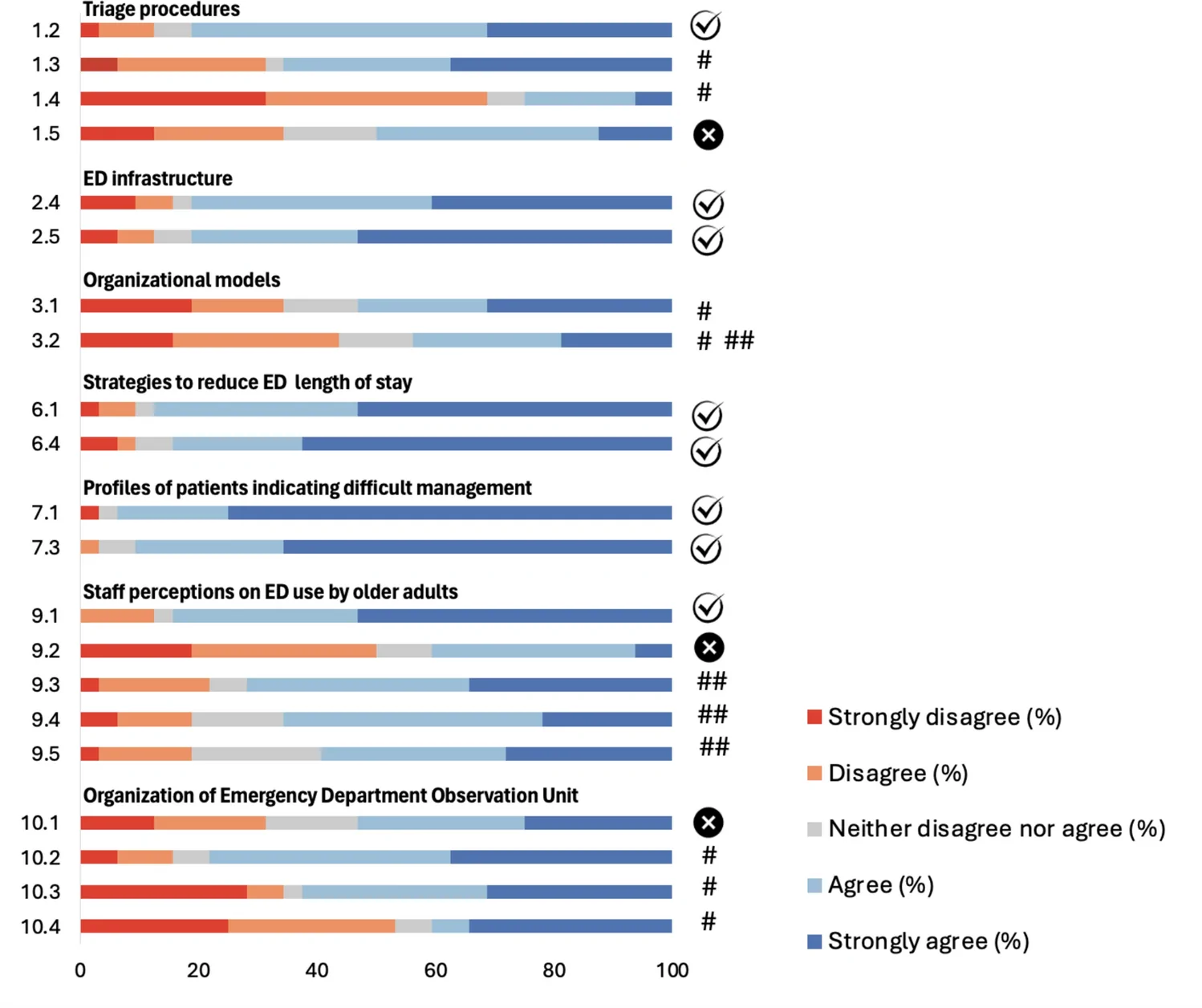
Likert scale responses by item (Round 2). Symbols indicate the level of consensus: a checkmark denotes full consensus across all groups; a black cross indicates a lack of consensus globally and within subgroups of geriatricians and emergency physicians. A single hashtag (#) denotes consensus among geriatricians only, while a double hashtag (##) consensus among emergency physicians only. The presence of both # and ## indicates consensus within each subgroup but in opposing directions

After Round 2, overall agreement increased to 74% (37 items), with further consensus achieved in Statements 2, 6, and 7 (Fig. 2). These included: (i) architectural modifications in the ED tailored to frail older adults, including dedicated waiting/stay areas, caregiver-friendly spaces, movable partitions, and geriatric admission rooms (81.3–100%); (ii) organizational improvements, such as increasing bed availability for frail patients, predefined clinical pathways, access to on-call or dedicated geriatric expertise, and geriatric admission areas (up to 93.8%); (iii) patient profiles associated with higher management complexity, including age > 85 years, elevated social needs, nursing homes residence, and cognitive, behavioral, or severe motor impairments.

Conversely, no full consensus emerged for Statements 1, 3, 9, and 10.

As for Statement 1, agreement was reached on the need to train triage nurses in the principles of geriatrics (96.9% in Round 1) and to implement the Silver Code alongside traditional triage coding systems (81.3% in Round 2). However, no consensus was reached on the usefulness of directing most patients to geriatric fast-track pathways (65.6%), the use of color-coded triage categories as a stand-alone approach to determine priority of access (68.8%), or the adoption of the Emergency Severity Index (ESI) for severity assessment (50.0%). Disagreement mainly reflected divergent views between geriatricians and emergency physicians (particularly regarding color-coded triage and geriatric fast-track pathways), whereas the ESI result suggested broader uncertainty across panelists.

As for Statement 3, consensus was achieved on the importance of staff training in geriatric care (100%), developing a dedicated “frailty unit” within the ED (90.6%), and promoting continuous caregiver presence (93.8%). However, emergency physicians tended to favor an on-call model of geriatrician involvement, whereas geriatricians supported permanent on-site presence, precluding consensus on the preferred model of geriatrician involvement.

As for Statement 9, panelists agreed that older adults attend the ED more frequently than younger patients (84.4%), but views diverged on the appropriateness of such use. This lack of consensus largely reflected inter-specialty differences. Emergency physicians more often viewed a substantial proportion of older adults’ ED visits as potentially inappropriate and contributory to overcrowding, noting that ED resources should primarily be directed toward critically ill patients rather than presentations related to chronic conditions. By contrast, geriatricians generally considered these visits appropriate, emphasizing that ED resources should also be allocated to older patients presenting with acute exacerbations of chronic conditions.

As for Statement 10, consensus was reached that EDOUs should adopt a multidisciplinary approach (87.5%). However, disagreement persisted regarding the creation of dedicated geriatric sections and routine co-management, as each specialty tended to consider the other’s routine involvement unnecessary or not appropriate for EDOU care.

## Discussion

This two-round Delphi study, involving 32 clinicians (geriatricians and emergency physicians) from two Italian academic societies (AcEMC and SIGG), identified shared priorities to improve care for older adults within EDs in Italy.

Consensus was achieved for 37 of 50 items (74%), indicating broad agreement on key clinical (Statements 4, 5, 7, 8), structural (Statement 2), and organizational (Statement 6) adaptations. Areas of disagreement concerned triage models (Statement 1), mode of geriatrician involvement (Statements 3, 10), and staff perceptions on ED use by older adults (Statement 9). Overall, these findings reflect strong inter-specialty commitment to improving geriatric emergency care, while highlighting areas of divergence that require further evaluation in the Italian context.

The areas of agreement—including the importance of early frailty detection at triage, the provision of multidisciplinary care, and the development of adapted environments—closely mirror priorities already identified in the European guidance on geriatric emergency care [15]. Panelists also recognized the need for the early identification of delirium and other atypical presentations of acute illness in older adults, consistent with international recommendations from geriatric ED models [26, 27]. In addition, they emphasized the importance of implementing preventive and management strategies, such as hydration, mobility support, falls prevention, and medication review, in line with international pilot ED interventions [28, 29].

Older-friendly environmental adaptations (e.g., dedicated areas for older patients and caregivers) were also endorsed by most panelists. This likely reflects a shared recognition of everyday challenges within ED spaces—overcrowding and high levels of noise and visual stimulation—which may be particularly detrimental for older adults. It also highlights the practical constraints many EDs face in enabling caregivers to remain at the bedside, underscoring an important implementation target for improving care [29, 30].

Divergent perceptions of “inappropriate” ED use among older adults warrant cautious interpretation. Although some emergency physicians viewed many visits as potentially inappropriate—consistent with prior literature [31, 32]—this pattern may reflect structural gaps in the Italian healthcare system rather than patient misuse. International evidence points to contributing factors such as limited alternatives to hospitalization and fragmented post-acute care pathways [33, 34]. Consistently, Italian data suggest that many ED presentations among older adults are driven by frailty and discontinuity of care, highlighting the need to strengthen hospital–community integration [5].

Additional disagreement—regarding geriatric fast-track pathways, triage coding approaches, and the models of geriatrician involvement in the ED (on-site vs on-call)—also appears to reflect organizational and feasibility constraints rather than differences in clinical principles.

While these approaches are widely regarded as desirable, implementation remains challenging in high-pressure ED settings. Contributing factors likely include uneven access to geriatric services across hospitals, workforce shortages, limited time for triage assessments, and uncertainty about the scalability of dedicated pathways in routine workflows [5, 13, 35]. These divergences have important implications for implementation and may limit translation into practice unless addressed through explicit escalation criteria and locally adaptable care models.

Taken together, these findings highlight a persistent disconnect between the recognized value of geriatric-oriented models and their adoption in routine national practice. A recent review on emerging themes and future directions in Geriatric Emergency Departments (GEDs) highlights the need for “calls to action” to support the integration of these care models within health systems, particularly through strengthened interdisciplinary collaboration and dedicated organizational pathways for older patients—both central themes in our study [36].

**Fig. 3:**
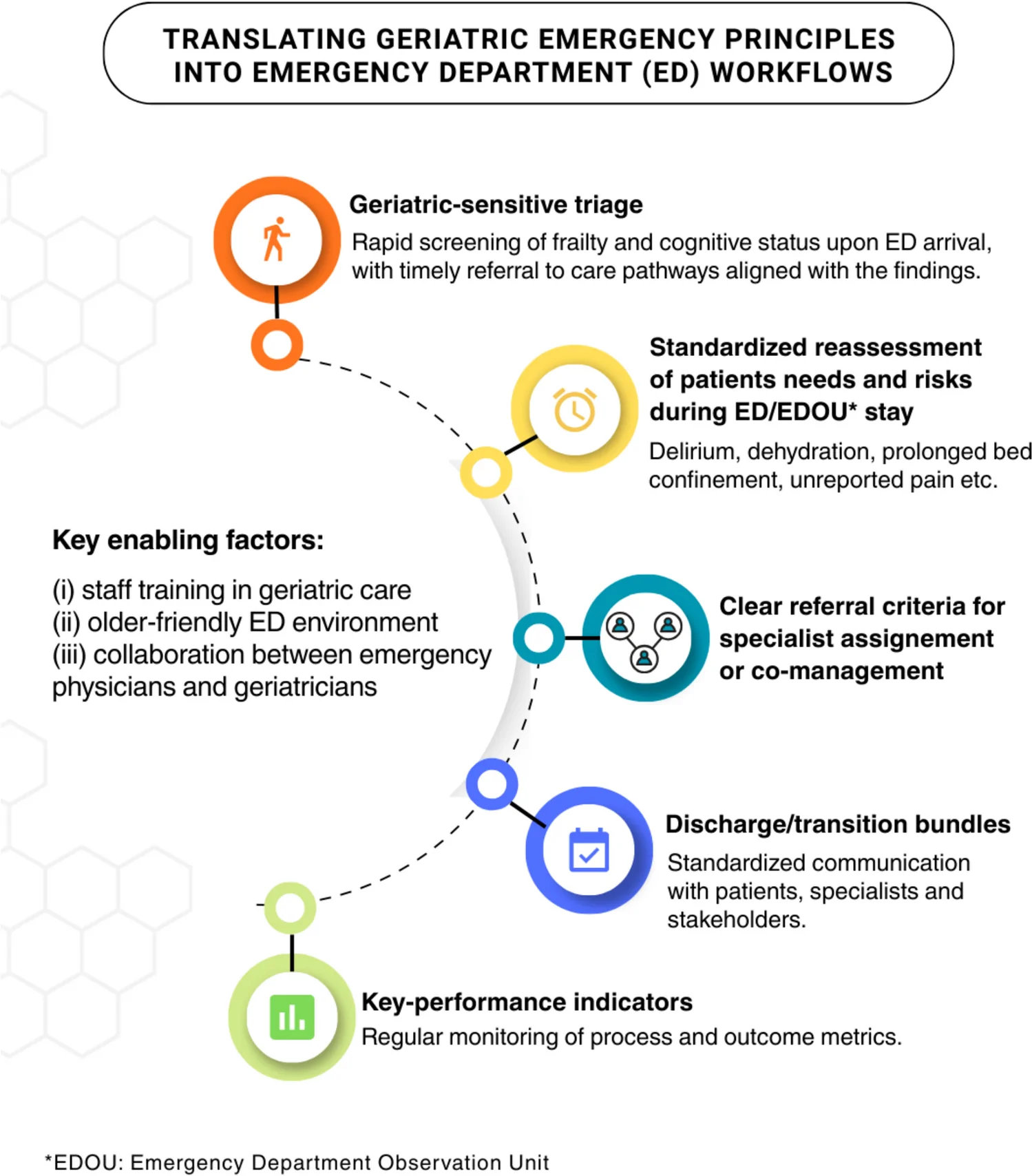
Context-sensitive implementation roadmap for geriatric emergency care in Italy

To facilitate translation into practice, consensus priorities can be operationalized across five key implementation domains, supported by (i) staff training in geriatric care, (ii) older-friendly ED environment, and (iii) structured collaboration between emergency physicians and geriatricians (Fig. 3).

1. Geriatric-sensitive triage: systematic use of brief validated tools to detect vulnerability (e.g., Identification of Seniors at Risk, ISAR [37], delirium risk assessment tool [38]) to trigger timely referral to appropriate care pathways;
2. Standardized reassessment of patients’ needs and risks during ED/EDOU stay (e.g., delirium, dehydration, prolonged bed confinement, unreported pain);
3. Clear referral criteria for specialist assignment or co-management;
4. Discharge/transition bundles: standardized communication with patients, specialists, and stakeholders;
5. Key-performance indicators: process and outcome metrics to monitor uptake and impact.

These principles are not intended to be mandatory; they provide a scalable framework that can be adapted to heterogeneous Italian settings, where local experiences exist but remain unevenly embedded within the National Health Service.

This study is the first national inter-specialty consensus on geriatric emergency care in Italy, developed through a rigorous Delphi methodology and involving balanced participation from geriatricians and emergency physicians across the country. However, several limitations should be acknowledged. First, the Delphi method reflects expert judgment and is inherently influenced by panel composition and item framing. Second, although national in scope, the panel may not fully represent all Italian ED contexts; large academic centers may be over-represented, while smaller peripheral hospitals may be under-represented. Third, this study did not include empirical validation of the proposed interventions or assessment of patient-level outcomes. Future studies are needed to evaluate the clinical impact and scalability of these findings in the Italian context.

## Conclusions

This national Delphi consensus defines shared priorities between emergency physicians and geriatricians for geriatric emergency care in Italy and identifies inter-specialty divergences relevant to implementation. Rather than weakening the findings, these divergences highlight areas requiring context-adapted solutions—particularly in triage and staffing models, including clear definitions of geriatricians’ roles within ED workflows. The consensus offers a framework for geriatric-oriented ED pathways, to be implemented through phased, resource-sensitive strategies, supported by measurable process indicators and prospective evaluation.

## Supplementary Information

Below is the link to the electronic supplementary material.

Supplementary File 1 (Online Resource S1–S2) (DOCX 63 kb)

## Data Availability

Data can be made accessible for statistical and scientific research under certain conditions. For further information, please contact the corresponding author.
