# Supplementary material for: Caring for older adults in the Emergency Department: a Delphi-based national consensus by the Academy of Emergency Medicine and Care and the Italian Society of Gerontology and Geriatrics
Source: Eur Geriatr Med. 2026 May 3;17(4):1991–2001. doi: 10.1007/s41999-026-01462-6 (PMC13562276; doi:10.1007/s41999-026-01462-6)
Supplement: Supplementary file 1 [file 41999_2026_1462_MOESM1_ESM.docx]

**Caring for Older Adults in the Emergency Department: A Delphi-Based National Consensus by the Academy of Emergency Medicine and Care and the Italian Society of Gerontology and Geriatrics.**

**Authors:**

Maria Cristina Ferrara^a,b^*, Eleonora Cucini^a^*, Martina Marelli^a^, Antonella Zambon^c,d^, Andrea Ungar^e^, Lorenzo Ghiadoni^f^, Antonio Cherubini^g,h^, Michele Cosimo Santoro^i^, Alessandra Marengoni^j,k^, Ciro Paolillo^l^, Ivo Casagranda^m #^, Giuseppe Bellelli^a,n^ ^#^ (Annex 1)

**Affiliations:**

a School of Medicine and Surgery, University of Milano-Bicocca, Milan, Italy
b Centro Studi Dipartimentale sulla Medicina della Complessità e Cure Palliative Virgilio Floriani, University of Milano-Bicocca, Monza, Italy
c Department of Statistics and Quantitative Methods, University of Milano-Bicocca, Milan, Italy
d Biostatistics Unit, IRCCS Istituto Auxologico Italiano, Milan, Italy
e Geriatric and cardio-geriatric medicine, Department of Clinical and Experimental Medicine, University of Florence and Careggi Hospital, Florence
f Department of Clinical and Experimental Medicine, University of Pisa, Pisa, Italy
g Department of Clinical and Molecular Sciences, Università Politecnica delle Marche, Ancona, Italy
h Geriatria, Accettazione geriatrica e Centro di Ricerca per l’invecchiamento, IRCCS INRCA, Ancona, Italy
i Emergency Department, Foundation Polyclinic University A. Gemelli, Rome, Italy
j Department of Clinical and Experimental Science, University of Brescia, Brescia, Italy
k Aging Research Center, NVS Department, Karolinska Institutet, Stockholm, Sweden
l Emergency Department, Ospedale Civile Maggiore AOUI Verona, Italy
m Academy of Emergency Medicine and Care (AcEMC), Pavia, Italy
n Acute Geriatric Unit, Fondazione IRCCS San Gerardo dei Tintori, Monza, Italy

* These authors equally contributed as co-first authors

\# These authors equally contributed as co-senior authors

**Supplementary material**

**Online Resource S1:** Expert panel’s composition

**Online Resource S2:** Delphi questionnaire on the management of older patients in the Emergency Department (ED).

**Online Resource S1** Expert panel’s composition

| **Variable** | **N (%)** |
| --- | --- |
| **Specialty** |  |
| ***Geriatricians*** | **16 (50)** |
| ***Emergency physicians*** | **16 (50)** |
| **Sex, male** | **23 (72)** |
| **Age** |  |
| ***< 40*** | **1 (3)** |
| ***41-50*** | **5 (16)** |
| ***51-60*** | **15 (47)** |
| ***61-70*** | **11 (34)** |
| **Geographic area** |  |
| ***Northern Italy*** | **12 (37.5)** |
| ***Center Italy*** | **15 (47)** |
| ***Southern Italy*** | **5 (15.5)** |

**Online Resource S2** Delphi questionnaire on the management of older patients in the Emergency Department (ED)

*The questionnaire consists of 10 statements and their related items, addressing both organizational/structural aspects as well as clinical and preventive issues in the management of older adults in the Emergency Department (ED).*

*Each statement consists of 5 items. Respondents are asked to rate each item using a 5-point Likert scale: 1) Strongly disagree; 2) Disagree; 3) Neither agree nor disagree; 4) Agree; 5) Strongly agree.*

*Consensus on the items is considered achieved when at least 80% of experts express agreement (Likert 4 or 5) or disagreement (Likert 1 or 2). At the end of each statement, it is possible to provide open comments.*

*By answering these questions, you give your consent to participate in this consensus process. Your responses will be treated with the utmost confidentiality, and the data collected will be anonymous, not attributable to you in any way.*

*We sincerely thank you for your time and effort in helping to ensure the highest quality of care for older adults in the Emergency Department.*

### **Statement 1**

### Triage should be structured to consider the characteristics of older patients

**Items**

1. Provide specific training on older adults for nurses trained to perform triage

2. Assign all older adults a Silver Code in addition to the traditional triage code

3. Direct most patients to a fast-track geriatric pathway*

4. Use the “color code” as the sole criterion to determine priority of access**

5. Use a triage protocol such as Emergency Severity Index (ESI^1^)***

^1^ The Emergency Severity Index (ESI) is a triage tool used to classify patients based on the severity of their conditions and the need for immediate intervention. The ESI assigns a score from 1 to 5, where 1 indicates a critical situation requiring immediate intervention and 5 a less urgent condition. This tool helps quickly determine which patients need urgent care and allows for efficient allocation of resources, reducing waiting times.

*Additional interpretative note provided after the first round:*

**1.3 This sentence suggests that most older patients should be directed to a specific and rapid geriatric assessment and management pathway to implement a personalized approach according to their clinical needs and level of frailty.*

***1.4 This sentence suggests using only the traditional color code system to establish the priority of access to care, regardless of the patient’s characteristics.*

**** 1.5 This sentence suggests adopting a protocol such as the Emergency Severity Index (ESI) as a triage tool to classify patients based on the severity of their clinical condition and the need for immediate intervention. This system allows rapid identification of higher-priority patients and efficient allocation of resources, reducing waiting times.*

### **Statement 2**

### ED architecture should be suitable for the needs of older patients

**Items**

1. Provide a waiting/stay area properly equipped for the frailest patients^2^

2. Provide space for caregiver presence

3. Provide at least movable partitions

4. Provide an area near the general ED

5. Ensure the presence of a geriatric admission room^3^

^2^ Frailty is defined as an age-related clinical condition of a syndromic nature, characterized by reduced functional reserves and a decreased capacity to withstand stress of any kind (both exogenous and endogenous), resulting from the cumulative decline of multiple physiological systems. Frailty increases the risk of adverse events such as falls, disability, hospitalization, and mortality.

^3^ Admission room is defined as an organized space that hosts patients coming from the Emergency Department while they wait to be assigned to the ward most appropriate for their clinical problems.

### **Statement 3**

### The ED should be organized to provide specific management for older patients

**Items**

1. Ensure a fixed presence of a geriatrician in the ED*

2. Ensure geriatrician presence only “on call” for the emergency physician**

3. Train emergency physicians and nurses in managing older patients

4. Establish a “frailty unit” with a multidisciplinary approach (emergency physician and nurse, geriatrician, social worker) operating permanently in the ED

5. Promote constant caregiver presence in the ED

*Additional interpretative note provided after the first round:*

** 3.1 This sentence suggests the need to guarantee the constant presence of a geriatrician in the ED to improve care for older patients, who may require specific attention to their health conditions.*

*** 3.2 This sentence suggests that a geriatrician can be called to intervene, when necessary, at the request of the emergency physician. This model provides geriatric consultation to support the emergency team, limited to cases where specialized geriatric intervention is deemed essential.*

### **Statement 4**

### In the general clinical management of older adults in the ED, some priorities should be considered

**Items**

1. Consider situations that may indicate a risk of deterioration

2. Highlight clinical conditions at risk of deterioration with atypical presentation symptoms

3. Early identification of patients with delirium using dedicated screening tests

4. Systematically assess possible adverse drug reactions

5. Systematically assess the presence of disability and frailty

### **Statement 5**

### Interventions should be implemented to reduce the risk of a negative outcome

**Items**

1. Provide specific protocols for hydration and nutrition of older adults during their stay in the ED

2. Systematically identify patients with delirium using specific screening tools

3. Implement measures to counteract immobilization

4. Implement protocols for fall prevention in at-risk patients

5. Use pain assessment tools specific for patients with dementia

### **Statement 6**

### Interventions should be implemented to reduce the length of stay of older adults in the ED

**Items**

1. Increase hospital beds for frail older adults affected by acute conditions

2. Develop specific clinical pathways for older adults

3. Ensure the presence of a geriatrician in the ED or on call

4. Use the geriatric admission room

5. Activate care pathways already agreed with the TOC^4^ from the ED

^4^ TOC (Territorial Operations Center) is a territorial health organizational service, described in the recent Ministerial Decree 77, aimed at coordinating and integrating the various health and social services present in the territory, with particular attention to continuity of care.

### **Statement 7**

### Conditions that suggest overall difficult management in the ED

**Items**

1. Older adults > 85 years

2. Patients with socio-assistance issues

3. Patients coming from nursing homes / protected residences

4. Patients with significant cognitive deficits and/or behavioral disorders

5. Patients with severe motor impairment

### **Statement 8**

### Systematic actions should be implemented for proper management of older adults in the ED

**Items**

1. Identify frail and/or disabled patients using specific tools

2. Identify patients with delirium using specific tools

3. Identify patients with pre-existing cognitive deficits

4. Identify patients with socio-assistance issues

5. Identify patients with repeated ED visits

### **Statement 9**

### Staff perceptions on older adults’ use of the ED can be represented by the following statements

**Items**

1. Older patients visit the ED more frequently than younger patients

2. They are treated with the same intensity of care as younger patients*

3. ED use is often inappropriate**

4. They contribute to overcrowding***

5. ED staff should focus on managing critically ill patients, not older adults with chronic conditions****

*Additional interpretative note provided after the first round:*

** 9.2 This sentence suggests that, despite age differences, older adults receive a level of care intensity comparable to that of younger patients, reflecting an approach based on individual clinical needs rather than chronological age.*

*** 9.3 This sentence suggests that older adults often use the ED for situations that could be managed at home or in other healthcare settings (e.g., due to limited access to other resources or because the ED is perceived as the only available option).*

**** 9.4 This sentence suggests that older adults’ visits contribute to overcrowding in the EDt, which may be related to the high volume of older patients or difficulties in promptly redirecting them to other services.*

**** 9.5 *This sentence suggests that managing older adults with chronic conditions should not fall within the Emergency Department staff’s responsibility, which should be focused on acute emergencies and critical situations.*

### **Statement 10**

### The benefits of an Emergency Department Observation Unit (EDOU) include reduced ED length of stay for older adults, improved ED efficiency, and better clinical and organizational outcomes

**Items**

1. Older adults should be admitted exclusively to a dedicated EDOU

2. The EDOU should include a section reserved for older adults

3. The EDOU should also be managed by geriatricians

4. The EDOU should be managed by emergency physicians

5. The EDOU should be managed with a multidisciplinary approach (emergency physician + geriatrician)

*Additional interpretative note provided after the first round:*

** 10.1 This sentence suggests that older patients should be admitted to an EDOU specifically dedicated to them. This approach aims to provide targeted care for their needs, with appropriate staff and resources to manage the complexity of their conditions, avoiding overload in other ED areas and optimizing resource use.*

*** 10.2 This sentence suggests the need to organize the EDOU with a dedicated space for older patients. This section would allow more appropriate and specialized care that considers their specific needs and provide a safer, more suitable environment for this population group.*

**** 10.3 This sentence emphasizes the importance of involving geriatricians in managing the EDOU to ensure a specialist approach focused on older patients’ care.*

***** 10.4* *This sentence suggests that the EDOU should be managed solely by emergency physicians, without any management involvement from geriatricians.*
